# Pooled analysis of clinical outcome for EGFR TKI-treated patients with *EGFR* mutation-positive NSCLC

**DOI:** 10.1111/jcmm.12278

**Published:** 2014-08-06

**Authors:** Luis Paz-Ares, Denis Soulières, Joachim Moecks, Ilze Bara, Tony Mok, Barbara Klughammer

**Affiliations:** a Department of Medical Oncology, Instituto de Biomedicina de Sevilla (HUVR, US and CSIC) and Hospital Universitario Virgen del RocioSeville, Spain; b Département de Médecine, Service d'hémato-oncologie, Centre Hospitalier de l'Université de MontréalMontréal, QC, Canada; c Department Bio-Mathematics, BIOMCON GmbHMannheim, Germany; d Global Medical Affairs Oncology, F. Hoffmann-La Roche LtdBasel, Switzerland; e Department of Clinical Oncology, The Chinese University of Hong Kong, Prince of Wales HospitalHong Kong, China; f Biomarker Oncology, F. Hoffmann-La Roche LtdBasel, Switzerland

**Keywords:** epidermal growth factor receptor (EGFR), tyrosine-kinase inhibitor, erlotinib, gefitinib, non-small-cell lung cancer (NSCLC), mutation, first line, afatinib, icotinib

## Abstract

Patients with non-small-cell lung cancer (NSCLC) appear to gain particular benefit from treatment with epidermal growth factor receptor (EGFR) tyrosine-kinase inhibitors (TKI) if their disease tests positive for *EGFR* activating mutations. Recently, several large, controlled, phase III studies have been published in NSCLC patients with *EGFR* mutation-positive tumours. Given the increased patient dataset now available, a comprehensive literature search for EGFR TKIs or chemotherapy in *EGFR* mutation-positive NSCLC was undertaken to update the results of a previously published pooled analysis. Pooling eligible progression-free survival (PFS) data from 27 erlotinib studies (*n* = 731), 54 gefitinib studies (*n* = 1802) and 20 chemotherapy studies (*n* = 984) provided median PFS values for each treatment. The pooled median PFS was: 12.4 months (95% accuracy intervals [AI] 11.6–13.4) for erlotinib-treated patients; 9.4 months (95% AI 9.0–9.8) for gefitinib-treated patients; and 5.6 months (95% AI 5.3–6.0) for chemotherapy. Both erlotinib and gefitinib resulted in significantly longer PFS than chemotherapy (permutation testing; *P* = 0.000 and *P* = 0.000, respectively). Data on more recent TKIs (afatinib, dacomitinib and icotinib) were insufficient at this time-point to carry out a pooled PFS analysis on these compounds. The results of this updated pooled analysis suggest a substantial clear PFS benefit of treating patients with *EGFR* mutation-positive NSCLC with erlotinib or gefitinib compared with chemotherapy.

## Introduction

The identification of new molecular targets for the treatment of lung cancer has revolutionized treatment paradigms for this disease. Non-small-cell lung cancer (NSCLC) therapy has benefited from the discovery of the epidermal growth factor receptor (EGFR) as a key mediator of cell proliferation. Efforts to identify agents to target EGFR led to the development of the EGFR tyrosine-kinase inhibitors (TKIs), which target the tyrosine-kinase (TK) domain of the receptor. Two EGFR TKIs have been approved for use in NSCLC in Europe and North America, erlotinib and gefitinib. Erlotinib has shown efficacy for second- or third-line treatment of NSCLC [1], as maintenance therapy [2] and for the first-line treatment of *EGFR* mutation-positive disease [3,4]. Gefitinib has shown efficacy for the treatment of locally advanced or metastatic NSCLC with activating *EGFR* mutations [5–7].

Low mutation rate and low availability of tumour samples limited the sample size for most of the efficacy analyses of erlotinib or gefitinib in patients with *EGFR* mutation-positive tumours. This prompted a retrospective pooled analysis by Paz-Ares *et al*. in 2010 [8], which reinforced the evidence that the preferential use of EGFR TKIs in patients with *EGFR* mutation-positive tumours may be warranted. Several additional large, phase III studies have since reported data in *EGFR* mutation-positive populations, including the WJTOG/802 [9], NEJ002 [10], OPTIMAL [3] and EURTAC [4] studies. Other anti-EGFR agents are also under investigation. Afatinib (an irreversible HER-family blocker), dacomitinib (an irreversible TKI of EGFR, HER2 and HER4) and icotinib (an EGFR TKI) have shown activity in *EGFR* mutation-positive NSCLC [11–16]. To date, the datasets for these compounds remain relatively limited although the results of one phase III trial have been recently reported [11].

The molecular biology of the *EGFR* mutation was reviewed extensively in the previous pooled analysis publication [8]. Briefly, EGFR plays a role in the mediation of cell signalling by regulating proliferation, angiogenesis and apoptosis [17,18]. Ninety per cent of NSCLC *EGFR* mutations comprise a leucine to arginine substitution at position 858 in exon 21 (L858R) or various deletion mutations in exon 19 [19–23]. Epidermal growth factor receptor mutations alter the TK pocket of the receptor, enhancing its sensitivity to EGFR TKIs.

Epidermal growth factor receptor mutations are found in around 10% of NSCLC in Caucasians and 30% of NSCLC in East-Asians [22]. Correlations between *EGFR* mutation-positive status and clinical characteristics have been reported, however, with the mutations being more common in the tumours of never-smokers and females, and in adenocarcinomas [22,24,25]. This correlation is not exclusive and patients cannot be assumed to have *EGFR* mutation-positive disease based on clinical profile alone. Therefore, *EGFR* mutation testing is essential; currently the European Society for Medical Oncology guidelines indicate that *EGFR* mutation testing is recommended as standard in non-squamous NSCLC [26]. Tumour specimens from curative surgery or bronchial biopsy are the gold standard for testing, but less than one-third of patients are suitable for surgery [27] and bronchial biopsy is impractical for poorly accessible tumours. Cytological samples, such as fine-needle aspirates, bronchial brushings, serum, plasma, circulating tumour cells and pleural effusion samples have all been used for *EGFR* mutation testing, but are considered less reliable because of heterogeneity of tissue samples and sparse cellularity [28].

Genotyping of *EGFR* mutations can be accomplished by several techniques. Direct DNA sequencing *e.g*. pyrosequencing and dideoxy ‘Sanger’ sequencing, will reveal any mutation. Detection by PCR (*e.g*. PCR-fragment length analysis) or real-time quantitative PCR (qPCR) is routinely employed to detect known, pre-specified *EGFR* mutations [29]. Locked nucleic acid genotyping is also used. In the clinical setting, rapid diagnostic testing may be employed with real-time PCR kits, which detect a specific number of mutations. Sequencing is still required to detect the rarer mutations. This pooled analysis focuses primarily on studies that included patients with exon 19 or exon 21 mutated NSCLC; multiple techniques have proven efficacious at detecting these classical mutations with high specificity and variable levels of detection. Cases identified by Sanger sequencing or highly sensitive methods appear to respond similarly to EGFR TKI [3,4].

The increased number of studies that have examined the efficacy of the EGFR TKIs in patients with exon 19 or exon 21 mutated NSCLC provides an expanded dataset for analysis. This paper describes an updated literature search for clinical studies of erlotinib, gefitinib and other EGFR TKIs in patients with *EGFR* mutation-positive NSCLC, and reports the results of a pooled analysis of erlotinib, gefitinib and chemotherapy, with the aim of providing updated median pooled progression-free survival (PFS) values. This study should help to provide robust recommendations for the clinical management of patients in this important patient subset.

## Materials and methods

### Selection criteria

All prospective and retrospective studies that examined erlotinib, gefitinib, icotinib, afatinib or dacomitinib as single-agent therapy or chemotherapy as single- or multiple-agent treatment for patients with *EGFR* mutation-positive NSCLC were eligible for inclusion. The studies were not critically assessed for methodology of *EGFR* mutation status determination.

### Literature search strategy

The literature was reviewed to identify studies for inclusion in the pooled analysis. The Datastar Web search engine was used to search Medline, BIOSIS Previews and Embase. The search date was 14 November 2011 and the search string used was (‘epidermal growth factor’ or ‘EGFR’) AND (‘lung’ OR ‘NSCLC’) AND (‘mutation’ OR ‘mutations’). Congress abstracts were searched, also on 14 November 2011, by using the same search string. The congresses searched were: the American Society of Clinical Oncology (ASCO) 2009–2011; the World Congress on Lung Cancer (WCLC) 2009–2011; and the European Cancer Organisation-European Society for Medical Oncology (ECCO-ESMO) 2009–2011. The references used in the original pooled analysis [8] were checked for updates (*e.g*. updated analyses or full papers). Only English language papers from 2004 or later were included. The papers were initially filtered by manually scanning the titles. Abstracts of the remaining papers were reviewed and filtered further. The remaining papers were then reviewed in full. The search excluded studies where: the results were only given graphically; two TKIs were given in sequence; PFS data for EGFR TKIs were presented as a class and not split by drug; patients were treated in the maintenance or adjuvant setting; EGFR TKI or chemotherapy was used in combination with any other therapy (including surgery); the PFS/time-to-progression (TTP) was not given for *EGFR* mutation-positive NSCLC; information was ambiguous; only updates were reported; *EGFR* mutation status analysis was performed with blood samples; results were presented for patients only with ‘other’ EGFR mutations (*i.e*. non-exon 19 deletion or L858R mutation); or patients received a non-standard dose. The search also excluded studies where patients were selected for *EGFR* mutation status plus: another biomarker; specific types or patterns of metastases; having very poor performance status (3+); having benefited from an EGFR TKI previously (including long-term responders). Individual case studies were excluded.

### Data extraction

The chosen end-point was PFS. For the purposes of the pooled analysis, TTP was considered equivalent to PFS. Where both PFS and TTP were reported, PFS was used in preference to TTP. The PFS/TTP data were extracted from the report by one individual and entered onto a database. Entries were validated by at least one other analyst. All references were checked for duplication; only the most recent publication was used for data (with verification against prior publications).

### Statistical analysis

The methodology was as described in Paz-Ares *et al*. [8], and is reported only briefly here. Individual data points were not available for PFS/TTP, therefore calculation of a pooled median PFS was made from the study medians. In some studies, a median PFS was not available, and simplifying assumptions (exponential distribution) were made to approximate the study median. In one study, a mean PFS value instead of median PFS was reported; based on the simplifying assumption of an underlying exponential distribution the median is simply the mean multiplied by ln(2). In some studies PFS was reported as ‘after *T*(time) months a fraction of the patients were without progression….’. The median PFS was estimated based on the assumptions of an exponential distribution by *T*/{ln(PFS) ln(x)}. Finally, in some cases multiple reports of PFS by time and percentage were reported. Here, the average of the multiple medians was taken to approximate the overall median in this study. The pooled median PFS (MPFS_all_) was then obtained by a weighted average of the single study medians, which was calculated by multiplying the study median (MPFS_(*i*)_) by the size of the study and summing over all studies. The result was divided by the total number of patients in all the studies (*N*_all_):


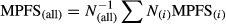


Valid confidence intervals (CIs) to assess inherent variation can only be calculated when individual data are given. These were not available for all studies so a surrogate ‘accuracy interval’ was calculated to reflect the comparative accuracy of the median estimates. The reported medians were treated as maximum likelihood estimates of the parameters of the exponential distribution to determine the pertaining confidence bands as ‘accuracy intervals’; the 90% and 95% confidence bands were used as surrogate ‘accuracy intervals’ (single studies and pooled median estimates, respectively).

Random permutations [30] across studies were generated for 20,000 runs to test the null hypothesis that there is no difference between treatments. This comparative test is statistically valid, but only refers to the given study pool (conditional test) and cannot be readily extrapolated to the total patient population.

The potential for publication bias was assessed by using funnel plots with the pertaining accuracy intervals.

### Sensitivity analysis

Sensitivity analyses were performed to assess the adequacy of the calculated accuracy intervals by using a resampling technique (‘bootstrap’) (*e.g*. Hesterberg *et al*. [30]) with 5000 runs.

## Results

### Breakdown of eligible studies

**Fig. 1:**
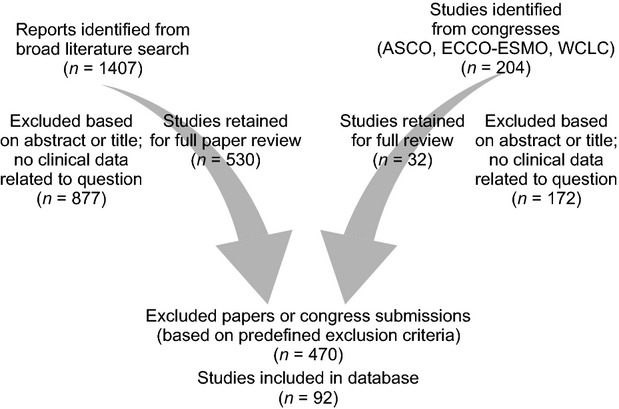
Breakdown of citations retrieved from literature searches and number of trials included in the analysis. ASCO: American Society of Clinical Oncology; ECCO-ESMO: European Cancer Organisation-European Society for Medical Oncology; WCLC: World Congress on Lung Cancer.

The number of studies identified and excluded is shown in Figure 1. In total, 92 papers or abstracts contained PFS values that were eligible for this pooled analysis. There were 26 studies that evaluated erlotinib, 54 studies that evaluated gefitinib, 20 that evaluated chemotherapy, two that evaluated icotinib and one that evaluated afatinib (Table 1). Of the 27 erlotinib studies, 10 were first line, 17 of 54 gefitinib studies were first line and 17 of 20 chemotherapy studies were first line. There were two erlotinib phase III trials, seven gefitinib phase III trials, nine chemotherapy phase III trials and one icotinib phase III trial. There were 10 retrospective erlotinib trials (*n* = 127), 26 retrospective gefitinib trials (*n* = 861), and 11 retrospective chemotherapy trials (*n* = 439). The total number of patients included in the pooled analysis was 3521 (731 were treated with erlotinib, 1802 were treated with gefitinib and 984 were treated with chemotherapy). Afatinib and icotinib were not included in the pooled analysis calculations, but studies were identified that included 129 afatinib-treated patients (US and Taiwanese) and 29 icotinib-treated patients (all Chinese). In studies where mutation types were reported individually the most common *EGFR* mutations were exon 19 deletions (53%) and L858R mutations (38%).

**Table 1:** Characteristics of the studies included in the pooled analysis to evaluate the effects of single-agent erlotinib, single-agent gefitinib or chemotherapy in patients with *EGFR* mutation-positive NSCLC (studies not included in the original analysis are highlighted)

Study	Design	Patients	Treatment	PFS/TTP
Erlotinib				
Ahn *et al*. [31]	Prospective	*n* = 24: Korean; ≥1 prior treatment; exon 19 deletion (*n* = 17); L858R (*n* = 5); exon 20 mutation (*n* = 1); exon 18 mutation and exon 19 deletion (*n* = 1)	Erlotinib 150 mg/day	TTP: 8.6 months
Jackman *et al*. [32]	Ph II, single-arm	*n* = 9: primarily white; chemo-naїve; ≥70 years; exon 19 deletion (*n* = 3); L858R (*n* = 5); L861Q and exon 19 deletion (*n* = 1)	Erlotinib 150 mg/day	TTP: 13 months
Jackman *et al*. [33]	Ph II, single-arm	*n* = 33: female; chemo-naїve; adenocarcinoma; *EGFR* mutations	Erlotinib 150 mg/day	TTP: 12.6 months
Miller *et al*. [34]	Ph II, single-arm	*n* = 18: BAC and adenocarcinoma, BAC subtype; 0–1 prior treatments; exon 19 or 21 *EGFR* mutation	Erlotinib 150 mg/day	PFS: 13 months
Pirker *et al*. [35]	Prospective (TRUST study)	*n* = 11: primarily white; chemo-naïve or previously treated; exon 19 deletion (*n* = 7); L858R (*n* = 5)	Erlotinib 150 mg/day	PFS: 405 days
Riely *et al*. [36]	Retrospective	*n* = 12: primarily white; chemo-naїve or previously treated; exon 19 deletion (*n* = 8); L858R (*n* = 4)	Erlotinib 150 mg/day	PFS: 12 months
Rosell *et al*. [37]	Prospective, Ph II	*n* = 12: Spanish; non-squamous cell carcinoma; exon 19 or 21 *EGFR* mutation	Erlotinib 150 mg/day	PFS: 13 months
Zhou *et al*. [38]	Retrospective	*n* = 6: Chinese; ≥1 previous treatment; *EGFR* mutations	Erlotinib 150 mg/day	TTP: 15.8 months
Amann *et al*. [39]	Retrospective; Ph II single-arm	*n* = 3: primarily white; mainly chemo-naïve; L858R (*n* = 3)	Erlotinib 150 mg/day	TTP: 13.1 months
Ciuleanu *et al*. [40]	Ph III; randomized comparison *versus* chemotherapy (TITAN)	*n* = 7: predominantly white; previously treated; *EGFR* mutation	Erlotinib 150 mg/day	PFS: 8.4 months
Choi *et al*. [41]	Retrospective; Ph II; single-arm	*n* = 21: Korean; chemo-naïve; exon 19 or exon 21 mutation	Erlotinib 150 mg/day	PFS: 11.5 months
De Greve *et al*. [42]	Prospective; Ph II; single-arm (FIELT)	*n* = 46: Belgian; chemo-naïve; exon 18 (*n* = 2), exon 19 (*n* = 27), exon 20 (*n* = 3), exon 21 (*n* = 15)	Erlotinib 150 mg/day	PFS rate at 3 months: 83% PFS rate at 6 months: 74% Median TTP not reached: 44+ weeks
Fiala *et al*. [43]	Retrospective	*n* = 9: Czech; *EGFR* mutation	Erlotinib 150 mg/day	TTP: 8.4 months
Rosell *et al*. [44]	Ph III; randomized comparison *versus* chemotherapy (EURTAC)	*n* = 86: primarily white; chemo-naïve; *EGFR* mutation	Erlotinib 150 mg/day	PFS: 9.7 months
Janne *et al*. [45]	Ph II; randomized comparison *versus* erlotinib plus chemotherapy (CALGB30406)	*n* = 33: primarily white; chemo-naïve; *EGFR* mutation	Erlotinib 150 mg/day	PFS: 14.1 months
Lynch *et al*. [46]	Ph II; randomized comparison *versus* erlotinib + bortezomib	*n* = 4: primarily white; mainly chemo-naïve; *EGFR* mutations	Erlotinib 150 mg/day	PFS: 4.3 months
Okano *et al*. [47]	Prospective; Ph II; single-arm	*n* = 10: Japanese; previously treated; *EGFR* mutation	Erlotinib 150 mg/day	PFS: 418 days (13.75 months)
Pallis *et al*. [48]	Prospective; Ph II; single-arm	*n* = 9: Greek; chemo-naïve; exon 19 deletion (*n* = 4); L858R (*n* = 5)	Erlotinib 150 mg/day	PFS: 12.4 months
Puente *et al*. [49]	Retrospective; single-arm	*n* = 23: chemo-naïve; *EGFR* mutation	Erlotinib 150 mg/day	PFS: 11 months
Rosell *et al*. [50]	Prospective; single-arm	*n* = 217: primarily white; chemo-naïve or previously treated; exon 19 deletion (*n* = 135); L858R (*n* = 82)	Erlotinib 150 mg/day	PFS: 14 months
Rotella *et al*. [51]	Retrospective; single-arm	*n* = 8: previously treated; exon 19 (*n* = 6); exon 21 (*n* = 2)	Erlotinib 150 mg/day	PFS: 18 months
Spigel *et al*. [52]	Prospective, ph II	*n* = 3: primarily white; 1–2 prior treatments; *EGFR* mutations	Erlotinib 150 mg/day	PFS: 9.23 months
Sun *et al*. [53]	Retrospective; single-arm	*n* = 35: Korean; mainly previously treated; exon 19 deletion or L858R	Erlotinib 150 mg/day	PFS: 8.0 months
Takahashi *et al*. [54]	Retrospective; Ph II; single-arm	*n* = 2; Japanese; chemo-naïve or previously treated; exon 19 deletion	Erlotinib 150 mg/day	TTP: Patient 1: 308 days (10.1 months) Patient 2: >973 days (>32 months)
Zhou *et al*. [3]	Ph III; randomized comparison *versus* carboplatin/gemcitabine (OPTIMAL)	*n* = 82: Chinese; chemo-naïve; exon 19 deletion (*n* = 43); L858R (*n* = 39)	Erlotinib 150 mg/day	PFS: 13.1 months
Zhu *et al*. [55]	Retrospective; single-arm	*n* = 8: Chinese; chemo-naïve or previously treated; exon 19 deletion (*n* = 5); L858R (*n* = 3)	Erlotinib 150 mg/day	PFS: 15.2 months
Gefitinib				
Asahina *et al*. [56]	Ph II, single-arm	*n* = 16: Japanese; chemo-naive; exon 19 deletion (*n* = 13); L858R (*n* = 3)	Gefitinib 250 mg/day	PFS: 8.9 months
Bell *et al*. [57]	Retrospective (Ph II IDEAL studies)	*n* = 14: ≥1 previous treatment; exon 19 deletion (*n* = 11); L858R (*n* = 2); InsG771 (*n* = 1)	Gefitinib 250 or 500 mg/day	TTP: 3.8 months
Buckingham *et al*. [58]	Retrospective	*n* = 17: ≥1 previous treatment; *EGFR* mutation	Gefitinib 250 mg/day	PFS: 13.6 months
Chou *et al*. [59]	Retrospective	*n* = 33: Taiwanese; prior platinum therapy; exon 18 substitution (*n* = 4); exon 19 deletion (*n* = 11); exon 20 substitution or deletion (*n* = 4); exon 21 substitution (*n* = 12); ≥1 mutation (*n* = 2)	Gefitinib 250 mg/day	PFS: 7.6 months
Cortes-Funes *et al*. [60]	Retrospective	*n* = 10: Spanish; ≥1 previous treatment; exon 19 deletion (*n* = 8); L858R (*n* = 2)	Gefitinib 250 mg/day	TTP: 12.3 months
D'Addario *et al*. [61]	Ph II, single-arm	*n* = 4: Swiss; chemo-naїve; exon 19 deletion (*n* = 2); L858R (*n* = 2)	Gefitinib 250 mg/day	TTP: 7.5 months
Dongiovanni *et al*. [62]	Retrospective	*n* = 9: Italian; chemo-naїve or previously treated; exon 19 deletion (*n* = 8); L858R (*n* = 1)	Gefitinib 250 mg/day	TTP: 14.9 months
Fukuoka *et al*. [63]	Ph III IPASS; randomized comparison with carboplatin/paclitaxel	*n* = 132: East-Asian; adenocarcinoma; never-smokers; chemo-naїve; *EGFR* mutation	Gefitinib 250 mg/day	PFS: 9.5 months
Han *et al*. [64]	Retrospective	*n* = 21: Korean; previously treated; exon 19 deletion (*n* = 12); L858R (*n* = 6); G719A (*n* = 3)	Gefitinib 250 mg/day	TTP: 13.8 months
Hirsch *et al*. [65]	Pooled analysis	*n* = 43: Italian or US; chemo-naїve or previously treated; exon 21 mutations (*n* = 31); exon 19 deletions (*n* = 11); mutations in exons 19 and 21 (*n* = 1)	Gefitinib 250 or 500 mg/day	PFS: 3 months
Ichihara *et al*. [66]	Retrospective	*n* = 30: Japanese; chemo-naїve and previously treated; exon 19 deletion (*n* = 16); L858R (*n* = 14)	Gefitinib 250 mg/day	PFS: 11.3 months
Inoue *et al*. [67]	Ph II, single-arm	*n* = 29: Japanese; chemo-naїve; poor performance status; exon 19 deletion (*n* = 18); L858R (*n* = 10), L861Q (*n* = 1)	Gefitinib 250 mg/day	PFS: 6.5 months
Inoue *et al*. [68]	Ph II, non-randomized comparison with standard chemotherapy	*n* = 16: Japanese; chemo-naїve; exon 19 deletion (*n* = 9); L858R (*n* = 7)	Gefitinib 250 mg/day	PFS: 9.7 months
Kim *et al*. [69]	Retrospective	*n* = 8: Korean; ≥1 previous treatment; exon 19 deletion (*n* = 5); L858R (*n* = 1)	Gefitinib 250 mg/day	TTP: 12.6 months
Kimura *et al*. [70]	Prospective, single-arm	*n* = 9: Japanese; chemo-naїve and previously treated; exon 19 deletion (*n* = 4); L858R (*n* = 4); V689L (*n* = 1)	Gefitinib 250 mg/day	PFS: 6.4 months
Kobayashi *et al*. [71]	Ph III, randomized comparison with carboplatin/paclitaxel	*n* = 98: chemo-naïve, *EGFR* mutation	Gefitinib 250 mg/day	PFS: 10.4 months
Koyama *et al*. [72]	Retrospective	*n* = 18: Japanese; chemo-naїve or previous treatment; G719C (*n* = 2); G719C and W731R (*n* = 1); P733S (*n* = 1); exon 19 deletion (*n* = 6); V738–I744 ins (*n* = 2); S768C (*n* = 1); T790M (*n* = 1); Q812R (1); V843I (*n* = 1); L858R (*n* = 2)	Gefitinib 250 mg/day	Mean TTP: 13.7 months
Massarelli *et al*. [73]	Retrospective	*n* = 7: Asian or Caucasian; chemo-naїve or previous treatment; exon 19 deletion (*n* = 6); G719A (*n* = 1)	Gefitinib 250 mg/day	TTP: 9.3 months
Oshita *et al*. [74]	Retrospective	*n* = 11: Japanese; ≥1 previous treatment; *EGFR* mutation	Gefitinib 250 mg/day	PFS: 16 months
Pallis *et al*. [75]	Retrospective	*n* = 11: Greek; ≥1 previous treatment; exon 19 deletion (*n* = 6); L858R (*n* = 3); G719D (*n* = 1); E746V (*n* = 1)	Gefitinib 250 mg/day	TTP: 14.7 months
Riely *et al*. [36]	Retrospective	*n* = 22: primarily white; chemo-naїve or previously treated; exon 19 deletion (*n* = 15); L858R (*n* = 7)	Gefitinib 250 mg/day	PFS: 12 months
Sequist *et al*. [76]	Ph II, single-arm	*n* = 31: primarily non-Asian; chemo-naїve; exon 19 deletion (*n* = 17); L858R (*n* = 8); atypical mutation (*n* = 6)	Gefitinib 250 mg/day	PFS: 9.2 months
Shao *et al*. [77]	Ph II, single-arm	*n* = 51: Taiwanese; chemo-naїve; *EGFR* mutation	Gefitinib 250 mg/day	PFS: 8.8 months
Shoji *et al*. [78]	Retrospective	*n* = 20; Japanese; chemo-naїve and previously treated; exon 19 deletion (*n* = 10); L858R (*n* = 8); E709A and G719S (*n* = 1); L858R and Y725Y (*n* = 1)	Gefitinib 250 mg/day	PFS: 14 months
Sugio *et al*. [79]	Ph II, single-arm	*n* = 19: Japanese; exon 19 deletion (*n* = 7); L858R (*n* = 10); exon 19 deletion and L858R (*n* = 1); exon 19 deletion and G796A (*n* = 1)	Gefitinib 250 mg/day	PFS: 7.1 months
Sunaga *et al*. [80]	Ph II, single-arm	*n* = 21: Japanese; chemo-naїve or previously treated; exon 19 deletion (*n* = 17); L858R (*n* = 4)	Gefitinib 250 mg/day	PFS: 12.9 months
Sutani *et al*. [81]	Ph II, single-arm	*n* = 27: Japanese; 0–1 previous treatments; exon 19 deletion; L858R, L861Q	Gefitinib 250 mg/day	TTP: 9.4 months
Takano *et al*. [82]	Retrospective	*n* = 85: Japanese; chemo-naїve or previously treated; exon 19 deletion (*n* = 49); L858R (*n* = 36)	Gefitinib 250 mg/day	PFS: 9.2 months
Tamura *et al*. [83]	Ph II, single-arm	*n* = 28: Japanese; 0–2 previous treatments; exon 19 deletion (*n* = 14); L858R (*n* = 14)	Gefitinib 250 mg/day	PFS: 11.5 months
Varella-Garcia *et al*. [84]	Retrospective	*n* = 27: Japanese; chemo-naїve or previously treated; *EGFR* mutations	Gefitinib 250 mg/day	TTP: 10.2 months
Xu *et al*. [85]	Retrospective	*n* = 32: Chinese; chemo-naїve and previous treatment; exon 19 deletion (*n* = 11); exon 19 – not deletion (*n* = 6); L858R (*n* = 6); exon 18 mutation (*n* = 6); exon 20 mutation (*n* = 2); exon 23 mutation (*n* = 1)	Gefitinib 250 mg/day	TTP: 15 months
Zhang *et al*. [86]	Retrospective	*n* = 12: Chinese; ≥1 previous treatment; exon 19 deletion (*n* = 4); L858 (*n* = 8)	Gefitinib 250 mg/day	PFS: 10 months
Asami *et al*. [87]	Prospective; Ph II; single-arm	*n* = 17: Japanese; exon 19 deletion (*n* = 7); L858R (*n* = 10)	Gefitinib 250 mg/day	PFS: 12.9 months
Azuma *et al*. [88]	Retrospective	*n* = 47: Japanese; chemo-naїve or previously treated; exon 19 deletion (*n* = 27); L858R (*n* = 20)	Gefitinib 250 mg/day	PFS: 6.7 months
Chen *et al*. [89]	Prospective, single-arm	*n* = 26: Chinese; chemo-naїve; *EGFR* mutation	Gefitinib 250 mg/day	PFS: 9.0 months
Chen *et al*. [90]	Prospective, Ph II; randomized comparison of gefitinib ± tegafur/uracil	*n* = 16: Taiwanese or Chinese; previously treated; exon 19 deletion (*n* = 12); L858R (*n* = 4)	Gefitinib 250 mg/day	PFS: 7.6 months
Douillard *et al*. [7]	Ph III INTEREST study; randomized, comparison with docetaxel	*n* = 19: primarily white; prior platinum chemotherapy; *EGFR* mutation	Gefitinib 250 mg/day	PFS: 7.0 months
Giovannetti *et al*. [91]	Retrospective, single-arm	*n* = 9: Italian; chemo-naїve or previously treated; exon 19 deletion (*n* = 7); L858R (*n* = 2)	Gefitinib 250 mg/day	TTP: 9.0 months
Inoue *et al*. [92]	Prospective; Ph II; single-arm (NEJ003)	*n* = 31: Japanese; chemo-naїve; *EGFR* mutation	Gefitinib 250 mg/day	PFS: 13.6 months
Lee *et al*. [93]	Ph III; randomized comparison with cisplatin/gemcitabine (First-SIGNAL)	*n* = 27: Korean; chemo-naïve; *EGFR* mutation	Gefitinib 250 mg/day	PFS: 7.9 months
Kim *et al*. [94]	Prospective; Ph II; single-arm	*n* = 45: Korean; chemo-naïve; exon 19 deletion (*n* = 29); L858R (*n* = 15); L861Q (*n* = 1)	Gefitinib 250 mg/day	PFS: 398 days (13.1 months)
Maemondo *et al*. [10]	Ph III; randomized comparison with carboplatin/paclitaxel (NEJSG002)	*n* = 114: Japanese; chemo-naïve; exon 19 deletion (*n* = 58); L858R (*n* = 49); Other (*n* = 7)	Gefitinib 250 mg/day	PFS: 10.8 months
Masago *et al*. [95]	Retrospective; single-arm	*n* = 47; Japanese; chemo-naїve or previously treated; *EGFR* mutation	Gefitinib 250 mg/day	PFS: 342 days (11.3 months)
Mitsudomi *et al*. [9]	Ph III; randomized comparison with cisplatin/docetaxel (WJTOG3405)	*n* = 86: Japanese; chemo-naïve; exon 19 deletion (*n* = 50); L858R (*n* = 36)	Gefitinib 250 mg/day	PFS: 9.2 months (8.4 months in stage IIIB/IV)
Moiseyenko *et al*. [96]	Prospective; single-arm	*n* = 25: Russian; chemo-naïve; exon 19 deletion (*n* = 17); L858R (*n* = 8)	Gefitinib 250 mg/day	PFS: 8.0 months
Park *et al*. [97]	Prospective, Ph II, single-arm	*n* = 3: Korean; previously treated; exon 19 deletion (*n* = 2); L858R (*n* = 1)	Gefitinib 250 mg/day	PFS: 5.8 months
Sun *et al*. [53]	Retrospective; single-arm	*n* = 42: Korean; mainly previously treated; exon 19 deletion or L858R	Gefitinib 250 mg/day	PFS: 11.9 months
Sun *et al*. [15]	Ph III; randomized comparison with icotinib (ICOGEN)	*n* = 39: Chinese; previously treated; *EGFR* mutation	Gefitinib 250 mg/day	PFS: 158 days
Uruga *et al*. [98]	Retrospective; single-arm	*n* = 9: Japanese; chemo-naїve or previously treated; exon 19 deletion (*n* = 6); L858R (*n* = 3)	Gefitinib 250 mg/day	PFS: 396 days
Wu *et al*. [99]	Retrospective; single-arm	*n* = 272: Taiwanese; chemo-naїve or previously treated; exon 19 deletion (*n* = 106); L858R (*n* = 114); other (*n* = 52)	Gefitinib 250 mg/day	PFS: 7.8 months
Wu *et al*. [100]	Retrospective; single-arm	*n* = 32: Chinese; previously treated; exon 19 deletion or L858R	Gefitinib 250 mg/day	PFS: 8.0 months
Yamaguchi *et al*. [101]	Retrospective; single-arm	*n* = 16: Japanese; chemo-naїve or previously treated; exon 19 deletion (*n* = 4); L858R (*n* = 8); exon 19 deletion + L858R (*n* = 4)	Gefitinib 250 mg/day	PFS: 246 days(8.1 months)
Yoshida *et al*. [102]	Prospective	Japanese; chemo-naïve (*n* = 23); exon 19 deletion or L858R Japanese; previously treated (*n* = 15); exon 19 deletion or L858R	Gefitinib 250 mg/day	PFS: 7.8 months PFS: 6.5 months
Chemotherapy				
Bell *et al*. [57]	Retrospective (Ph III INTACT studies; randomized comparison with gefitinib)	*n* = 9: primarily white; chemo-naїve; exon 19 deletion; L858R; other mutations	Paclitaxel/carboplatin or gemcitabine/cisplatin	PFS: 6.7 months
Eberhard *et al*. [103]	Retrospective (Ph III TRIBUTE study; randomized comparison with erlotinib plus carboplatin/paclitaxel)	*n* = 14: primarily white; chemo-naїve; exon 19 deletion; L858R; other mutations	Carboplatin/paclitaxel	TTP: 6.6 months
Fukuoka *et al*. [63]	Ph III; randomized comparison with gefitinib (IPASS)	*n* = 129: East-Asian; adenocarcinoma; never-smokers; chemo-naїve; *EGFR* mutation	Carboplatin/paclitaxel	PFS: 6.3 months
Inoue *et al*. [68]	Ph II; non-randomized comparison with gefitinib	*n* = 9: Japanese; chemo-naїve; exon 19 deletions (*n* = 8); L858R (*n* = 1)	Standard chemotherapy	PFS: 7.6 months
Lee *et al*. [104]	Retrospective	*n* = 17: Korean; chemo-naїve; patients receiving platinum-based chemotherapy; *EGFR* mutation	Platinum-based chemotherapy	TTP: 8 months paclitaxel, 9.7 months; gemcitabine, 7.4 months
Tambo *et al*. [105]	Retrospective	*n* = 26: Japanese; chemo-naïve; *EGFR* mutations	Chemotherapy	PFS: 8.4 months
Ciuleanu *et al*. [40]	Ph III; randomized comparison *versus* erlotinib (TITAN)	*n* = 4: predominantly white; previously treated; *EGFR* mutation	Docetaxel or pemetrexed (single-agent)	PFS: 9.9 months
Douillard *et al*. [7]	Ph III; randomized comparison with gefitinib (INTEREST)	*n* = 19; primarily white; prior platinum chemotherapy; *EGFR* mutation	Docetaxel	PFS: 4.1 months
Rosell *et al*. [44]	Ph III; randomized comparison *versus* erlotinib (EURTAC)	*n* = 87: primarily white; chemo-naïve; *EGFR* mutation	Platinum-based chemotherapy	PFS: 5.2 months
Kalikaki *et al*. [106]	Retrospective; single-arm	*n* = 9; Greek; chemo-naïve; *EGFR* mutation	Chemotherapy	TTP: 6.1 months
Kim *et al*. [107]	Retrospective	*n* = 67: Korean; chemo-naïve; *EGFR* mutation	Platinum-based chemotherapy	PFS: 7.1 months
Lin *et al*. [108]	Retrospective; single-arm	*n* = 56: East-Asian; chemo-naïve; primarily exon 19 deletion or L858R	Predominantly platinum-based chemotherapy	PFS: 6.1 months
Maemondo *et al*. [10]	Ph III; randomized comparison with gefitinib (NEJSG002)	*n* = 114: Japanese; chemo-naïve; exon 19 deletion (*n* = 59); L858R (*n* = 48); other (*n* = 7)	Carboplatin/paclitaxel	PFS: 5.4 months
Matsumoto *et al*. [109]	Retrospective; single-arm	*n* = 26: Japanese; chemo-naïve; *EGFR* mutation	Chemotherapy (50% platinum doublet, 50% non-platinum)	PFS: 6.9 months
Mitsudomi *et al*. [9]	Ph III; randomized comparison with gefitinib (WJTOG3405)	*n* = 86: Japanese; chemo-naïve; exon 19 deletion (*n* = 37); L858R (*n* = 49)	Cisplatin/docetaxel	PFS: 6.3 months (5.3 months in stage IIIB/IV)
Sun *et al*. [53]	Retrospective; single-arm	*n* = 67: Korean; chemo-naïve; exon 19 deletion or L858R	Chemotherapy	PFS: 5.1 months
Wu *et al*. [99]	Retrospective; single-arm	*n* = 93: Taiwanese; previously treated; exon 19 deletion (*n* = 43); L858R (*n* = 37); other (*n* = 13)	Pemetrexed monotherapy	PFS: 3.9 months
Wu *et al*. [100]	Retrospective; single-arm	*n* = 55: Chinese; chemo-naïve; exon 19 deletion (*n* = 32); L858R (*n* = 21); exon 19 deletion and L858R (*n* = 2)	Predominantly platinum-based chemotherapy	PFS: 4 months
Yoshida *et al*. [102]	Prospective	Japanese; chemo-naïve (*n* = 25) or previously treated (*n* = 20); exon 19 deletion or L858R	Cytotoxic chemotherapy	PFS: chemo-naïve: 5.1 months; previously treated: 4.0 months
Zhou *et al*. [3]	Ph III; randomized comparison *versus* erlotinib (OPTIMAL)	*n* = 72: Chinese; chemo-naïve; exon 19 deletion (*n* = 39); L858R (*n* = 33)	Carboplatin/gemcitabine	PFS: 4.6 months
Afatinib				
Yang *et al*. [110]	Ph II; randomized, single-arm	*n* = 129; Taiwanese and US; chemo-naïve, or one previous line of chemotherapy; exon 19 deletion (*n* = 52); L858R (*n* = 54); other (*n* = 23)	Afatinib 40 mg or 50 mg/day	PFS: 14 months
Icotinib				
Ren *et al*. [16]	Ph I; single-arm	*n* = 7: Chinese; previously treated; exon 19 deletion (*n* = 3); L858R (*n* = 4)	Icotinib (varied dose and schedule)	PFS: 141 days (4.6 months)
Sun *et al*. [15]	Ph III; randomized comparison with gefitinib (ICOGEN)	*n* = 27: Chinese; previously treated; *EGFR* mutation	Icotinib 125 mg three times/day	PFS: 198 days (6.5 months)

Ph: phase, BAC: bronchioloalveolar carcinoma.

There was a mixture of ethnicities included in the pooled analysis. Since only patients with *EGFR* activating mutations were included, no effect of ethnicity on efficacy was expected. Subgroup analyses revealed no striking differences between Asian and Caucasian patients with *EGFR* activating mutations and therefore, no adjustments were made in the analysis for ethnicity.

**Fig. 2:**
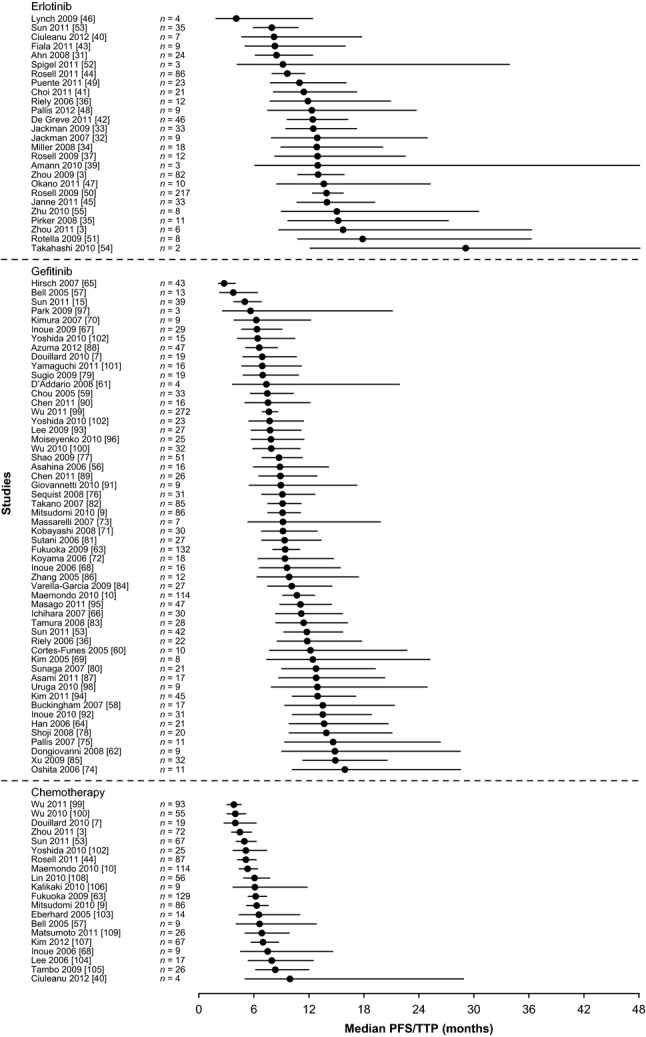
Forest plot showing analysis of median pooled PFS or TTP and 90% accuracy intervals during treatment with single-agent erlotinib, single-agent gefitinib or chemotherapy in all lines of treatment in patients with *EGFR* mutation-positive NSCLC. PFS: progression-free survival; TTP: time to progression; EGFR: epidermal growth factor receptor; NSCLC: non-small-cell lung cancer.

**Fig. 3:**
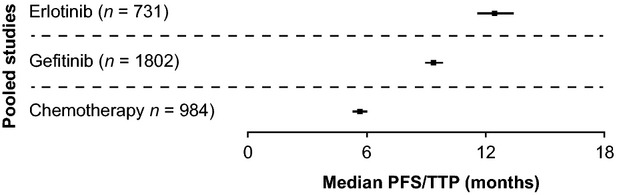
Forest plot showing pooled median PFS/TTP and 95% accuracy intervals with single-agent erlotinib, single-agent gefitinib or chemotherapy in patients with *EGFR* mutation-positive NSCLC in all lines of therapy. PFS: progression-free survival; TTP: time to progression; EGFR: epidermal growth factor receptor; NSCLC: non-small-cell lung cancer.

When the median PFS data are examined individually, there is a trend for erlotinib and gefitinib to report longer PFS times than chemotherapy (Fig. 2). This trend is confirmed when the pooled median PFS values are considered (Fig. 3). When analysed for significance by permutation testing (Table 2) there was a statistically significant increase in PFS for erlotinib compared with chemotherapy in the first line (*P* = 0.000), in lines other than first (*P* = 0.0022) and in all lines (*P* = 0.000). There was also a statistically significant increase in PFS for gefitinib compared with chemotherapy in the first line (*P* = 0.000), in lines other than first (*P* = 0.0039) and in all lines (*P* = 0.000). There were only three chemotherapy studies (*n* = 116) in treatment lines other than first; which limits the interpretation of this result, despite the significant *P*-value. However, the pooled median PFS value of 4.1 months for these three studies was not unexpected when the 5.8-month pooled median PFS for first-line chemotherapy-treated patients was considered.

**Table 2:** Pooled median PFS with 95% accuracy intervals for patients with *EGFR* mutation-positive tumours

Treatment	Number of studies	Pooled number of patients	Pooled median PFS	Accuracy interval	Bootstrap estimated 95% confidence limits
Any line of therapy					
Single-agent erlotinib	26	731	12.4	11.6–13.4	10.9–13.4
Single-agent gefitinib	54	1802	9.4	9.0–9.8	8.7–10.2
Chemotherapy	20	984	5.6	5.3–6.0	5.1–6.2
Predominantly first line*					
Single-agent erlotinib	10	354	12.0	10.8–13.3	10.8–13.1
Single-agent gefitinib	16	703	9.8	9.1–10.5	9.0–10.6
Chemotherapy	17	868	5.8	5.5–6.2	5.4–6.4
Lines of therapy other than first					
Single-agent erlotinib	17	377	12.9	11.6–14.3	10.0–13.9
Single-agent gefitinib	37	1099	9.2	8.6–9.7	8.3–10.5
Chemotherapy	3	116	4.1	3.5–5.0	n/a†

* Predominantly first line is ≥90% of patients treated in the first-line setting.

† Because of the low number of studies in this pool the bootstrap estimate is not trustworthy.

Comparing lines of treatment (Table 3), the pooled median PFS for chemotherapy was different when given as predominantly first-line treatment *versus* other lines of treatment (5.8 *versus* 4.1 months, respectively, *P* = 0.012). The analysis did not discriminate between single-agent chemotherapy and doublet chemotherapy. The pooled median PFS values for erlotinib and gefitinib were not statistically different between lines of treatment (erlotinib: 12.0 *versus* 12.9 months, for first and other lines, respectively, *P* = 0.678; gefitinib: 9.7 *versus* 9.1 months, for first and other lines, respectively, *P* = 0.283).

**Table 3:** Statistical analysis based on permutation testing

Study pool A	Study pool B	Differential median pooled PFS (A-B)	Estimated *P*-value (based on 20,000 random permutations)
Any line of therapy			
Chemotherapy	EGFR TKI	−4.7	0.000
Chemotherapy	Gefitinib	−3.8	0.000
Chemotherapy	Erlotinib	−6.8	0.000
Predominantly first line*			
Chemotherapy	EGFR TKI	−4.7	0.000
Chemotherapy	Gefitinib	−4.0	0.000
Chemotherapy	Erlotinib	−6.2	0.000
Lines of therapy other than first			
Chemotherapy	EGFR TKI	−6.1	0.0028
Chemotherapy	Gefitinib	−5.1	0.0039
Chemotherapy	Erlotinib	−8.8	0.0022
Chemotherapy			
Predominantly first line*	Lines of therapy other than first	1.7	0.012
Erlotinib			
Predominantly first line*	Lines of therapy other than first	−0.9	0.678
Gefitinib			
Predominantly first line*	Lines of therapy other than first	0.6	0.283

* Predominantly first line is ≥90% of patients treated in the first-line setting. This comparative test is statistically valid, but only refers to the given study pool (conditional test) and cannot be readily extrapolated to the total patient population.

Statistical analysis of a pooled median PFS could not be established for afatinib and icotinib because of lack of data. In one study for afatinib (*n* = 129), the PFS was 14 months [110], and in two studies (*n* = 27) and (*n* = 7) the PFS with icotinib was 6.5 months and 4.6 months, respectively [15,16] (Table 1). No eligible dacomitinib studies were identified at the time of analysis.

**Fig. 4:**
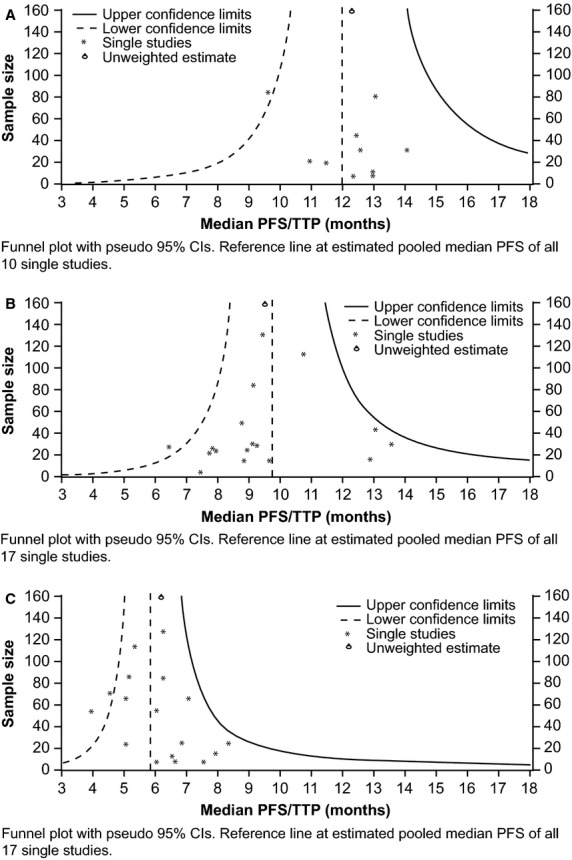
Funnel plots by using PFS/TTP as an outcome for (**A**) single-agent erlotinib (**B**) single-agent gefitinib and (**C**) chemotherapy in the first-line setting. PFS: progression-free survival; TTP: time to progression; CI: confidence interval.

Publication bias was assessed by using funnel plots with PFS/TTP as the outcome. These were symmetrical for each of the treatment groups (Fig. 4A–C).

## Discussion

The dataset analyzed here was almost double the size of that previously assessed [8]. The patient number was updated from 365 to 731 in the erlotinib arm, from 1069 to 1802 in the gefitinib arm and from 375 to 984 in the chemotherapy studies. Progression-free survival was again chosen as the end-point to assess. Because of high levels of crossover in post-study therapy, the use of overall survival as an end-point was not considered to be able to discern differences between treatments. This analysis indicates that PFS is longer in patients with *EGFR* mutation-positive NSCLC when treated with erlotinib (12.4 months) or gefitinib (9.4 months), compared with conventional chemotherapy (5.6 months). Permutation testing indicated that the difference in PFS was statistically significant, but this should be interpreted carefully, given that this significance applies to the current study pool and it cannot be readily extrapolated to the total patient population since a controlled randomized trial has not been carried out to confirm this. The bootstrap runs underlined the adequacy of the used accuracy intervals and thus provided confidence in the validity of the main analysis. The results are similar to those reported previously [8], in which statistically different PFS values for erlotinib (13.2 months) and gefitinib (9.8 months) were shown, as compared with chemotherapy (5.9 months).

As for the previous pooled analysis [8], there are limitations to this analysis. Statistical comparisons were made in this pooled retrospective analysis between erlotinib, gefitinib and chemotherapy based on PFS. Only high level information like median PFS was obtained from the publications, which could be used to calculate the pooled median PFS. In order to determine accuracy intervals, the simplifying assumption that PFS followed an exponential distribution was necessary but this was not verifiable. However, a bootstrap run confirmed the approximate validity of the accuracy intervals. Also, the schedule of visits for the progression of disease may have differed, according to the trial protocol. Furthermore, as the composition of the different patient groups (with respect to relevant risk factors) cannot be assessed, the results should be interpreted with due caution. However, there is no indication that the study pool and its treatment subgroups were not representative of the total patient population. Because of the comprehensive nature of the study pool, the differences reported have resulted from data collated from almost the entire body of evidence published up to November 2011. Furthermore, the line of treatment represents an important clinical risk factor that was considered as part of this study pool; the differences between treatments were confirmed through the treatment lines investigated, lending weight to the analyses. These points suggest that, despite the inherent limitations, the differences seen in the study pool deserve attention when the total patient population is considered. Because of the comprehensive approach to pool all available evidence, retrospective studies were included and no quality analysis of the data or mutation testing was possible. Additionally, this pooled analysis compares only median PFS values, and does not include other measures of response or safety/toxicity. Finally, PFS is not always assessed in the same way across all studies, a further source of variability.

The present analysis included large, phase III studies that prospectively evaluated the treatments in patients with *EGFR* mutation-positive NSCLC, which augment the dataset with robust data. The dataset reported here seems to be in agreement with the primary results of the additional phase III trials, which all reported significantly longer PFS with EGFR TKI therapy compared with chemotherapy [3,4,10,111]. The gefitinib data reported here are also in agreement with results of a recently reported phase IV study of gefitinib in Caucasian patients with *EGFR* mutation-positive NSCLC (*n* = 106), which observed a median PFS of 9.7 months [112].

There is a need for randomized trials among the EGFR TKIs to directly compare efficacy and toxicity. Trials are ongoing or recently completed, which should provide further data. For example, a randomized, open-label trial recently reported a longer PFS, but slightly more adverse events, with dacomitinib compared with erlotinib in patients with previously treated advanced NSCLC (*n* = 188) [113]. The LUX-Lung 7 study is a comparative study of afatinib *versus* gefitinib (NCT01466660) for *EGFR* mutation-positive NSCLC and is currently recruiting patients. Finally, CTONG 0901 (NCT01024413) was a randomized, phase II trial comparing first-line erlotinib with first-line gefitinib in patients with advanced NSCLC with exon 21 mutations; results have not yet been reported.

Afatinib and icotinib were included in the literature search and pooled analysis. However, there are currently very limited data on these two EGFR TKIs, and statistical analysis of a pooled median PFS could not be accomplished. Further clinical trials are required to establish the role of these agents in the treatment of patients with *EGFR* mutation-positive NSCLC. Recently, a phase III clinical trial of afatinib was completed and showed that patients receiving afatinib (*n* = 230) had a median PFS of 11.1 months (compared with 6.9 months with chemotherapy; hazard ratio [HR] = 0.58, 95% CI 0.43–0.78, *P* = 0.0004) [11]. This study was reported after the pooled analysis was complete, and is therefore not included in the analysis. In a subset of patients with exon 19 and L858R mutations, the median PFS was 13.6 months for afatinib, compared with 6.9 months for chemotherapy (HR = 0.47, 95% CI 0.34–0.65, *P* < 0.0001). Tolerability of anti-EGFR agents is also important; afatinib had relatively high levels of treatment-related adverse events (diarrhoea: 95%, leading to discontinuation in 1% of patients; rash: 62% and paronychia: 57%).

This pooled analysis utilizes data from a variety of ethnicities, ages and smoking histories, and includes both male and female patients. There are also a variety of *EGFR* mutations included, although the majority are exon 19 deletions and L858R mutations. The clear efficacy benefits across this range of clinical characteristics confirm the necessity of *EGFR* mutation testing, rather than reliance on clinical characteristics. Studies in which patients were ‘unselected’ or selected by clinical characteristics, demonstrate this. The First-SIGNAL study comparing the efficacy of single-agent gefitinib with gemcitabine plus cisplatin as first-line therapy for Korean ‘never-smokers’ with stage IIIB or IV lung adenocarcinoma not selected by *EGFR* mutation status [114] was unable to demonstrate superiority for gefitinib over chemotherapy. The IPASS study (phase III, *n* = 609, gefitinib or carboplatin plus paclitaxel in first line) showed non-inferiority of gefitinib to chemotherapy in non-smokers (or former light smokers) with adenocarcinoma. Only 60% of this preselected population had *EGFR* mutation-positive tumours. However, a significant PFS benefit of gefitinib compared with chemotherapy was reported in patients with established *EGFR* mutated-disease (9.5 months *versus* 6.3 months, respectively, HR = 0.48, 95% CI 0.36–0.64 *P* < 0.0001) [5]. Obviously, clinical characteristics are not an appropriate surrogate for *EGFR* mutation testing.

Furthermore, although common mutations, such as exon 19 deletions and L858R mutations in exon 21 have been associated with response to EGFR TKIs, many other mutations are detected only occasionally, and correlations with response are not defined. A recent study screened 681 cases and found 18 rare mutations; responses to EGFR TKIs were reported on a case by case basis and varied by mutation [115]. For example, exon 20 and 21 mutations were more likely to confer resistance to erlotinib or gefitinib, while exon 18 and 19 mutations were more often associated with improved efficacy outcome. An analysis of ‘other’ mutations from the SATURN, TRUST and TITAN trials suggested that some mutations (*e.g*. in exon 18) conferred a clinical benefit from erlotinib and others (*e.g*. in exon 20) had a prognostic influence on OS [116]. However, further data are needed.

Erlotinib and gefitinib also have differing responses in the common mutations, and new EGFR TKIs would be expected to also have differences. Exon 19 deletions and L858R mutations have shown similar *in vitro* sensitivity to gefitinib [22]; however, erlotinib and gefitinib have shown different clinical efficacy depending on whether exon 19 deletions and L858R mutations are present [117,118]. Despite these differences, both drugs have efficacy in patients with both of these mutations and these differences would not influence treatment selection.

As the number of clinical trials evaluating EGFR TKIs continues to increase, the number of patients eligible for pooled analyses such as this one will increase. Updating the dataset will enable more information to be gathered on the effect of TKIs on patients with NSCLC with rare mutations, as well as efficacy outcome of the newer agents. Assessing the benefit of different treatment regimens will also be important, for example, sequential intercalated chemotherapy and erlotinib is being investigated as a promising approach [119].

## Conclusions

A comprehensive review of PFS with EGFR TKI therapy or chemotherapy in the treatment of *EGFR* mutation-positive NSCLC was carried out, and included more than 3500 patients in a pooled analysis. The results demonstrate a clear PFS benefit of treating patients with *EGFR* mutation-positive NSCLC with an EGFR TKI compared with chemotherapy, with median pooled PFS values of 12.4 months (erlotinib), 9.4 months (gefitinib) and 5.6 months (chemotherapy) reported. This confirms that all patients should be tested for *EGFR* mutation status immediately on diagnosis of NSCLC, or as soon as feasible. This pooled dataset is in agreement with several large, prospective phase III studies that examined EGFR TKIs as first-line therapy, and strengthens the recommendation that *EGFR* mutation-positive NSCLC should be treated with erlotinib or gefitinib in the first line. If first-line therapy with EGFR TKIs is not achievable, then consideration should be given to treating with an anti-EGFR agent in any line of therapy, as PFS benefits over chemotherapy are obvious. Further trials should provide more insight into the role of second-generation EGFR TKIs for *EGFR* mutation-positive NSCLC.
